# A Review on Micro- to Nanocellulose Biopolymer Scaffold Forming for Tissue Engineering Applications

**DOI:** 10.3390/polym12092043

**Published:** 2020-09-08

**Authors:** H. P. S. Abdul Khalil, Fauziah Jummaat, Esam Bashir Yahya, N. G. Olaiya, A. S. Adnan, Munifah Abdat, Nasir N. A. M., Ahmad Sukari Halim, U. Seeta Uthaya Kumar, Rahul Bairwan, A. B. Suriani

**Affiliations:** 1 School of Industrial Technology, Universiti Sains Malaysia, Penang 11800, Malaysia; ngolaiya@futa.edu.ng (N.G.O.); seeta.uk@gmail.com (U.S.U.K.); 2 Management Science University Medical Centre, University Drive, Off Persiaran Olahraga, Section 13, Shah Alam 40100, Selangor, Malaysia; drazreenadnan@gmail.com; 3 CKD Resource Centre, School of Medical Sciences, Health Campus, USM, Kubang Kerian 16150, Kelantan, Malaysia; 4 Faculty of Medicine, Universitas Syiah Kuala, Banda Aceh 23311, Indonesia; munifahabdat_dr@unsyiah.ac.id; 5 Reconstructive Sciences Unit, School of Medical Sciences, Health Campus USM, Kubang Kerian 16150, Kelantan, Malaysia; nurazida@usm.my (N.N.A.M.); ashalim@usm.my (A.S.H.); 6 Department of Aeronautical engineering, School of Aeronautics, Neemrana 301705, Rajasthan, India; rahulbairwan94@gmail.com; 7 Nanotechnology Research Centre, Faculty of Science and Mathematics, UPSI, Tanjung Malim 35900, Perak, Malaysia; suriani@fsmt.upsi.edu.my

**Keywords:** nanocellulose, tissue engineering, scaffold, biomedical, biocompatible

## Abstract

Biopolymers have been used as a replacement material for synthetic polymers in scaffold forming due to its biocompatibility and nontoxic properties. Production of scaffold for tissue repair is a major part of tissue engineering. Tissue engineering techniques for scaffold forming with cellulose-based material is at the forefront of present-day research. Micro- and nanocellulose-based materials are at the forefront of scientific development in the areas of biomedical engineering. Cellulose in scaffold forming has attracted a lot of attention because of its availability and toxicity properties. The discovery of nanocellulose has further improved the usability of cellulose as a reinforcement in biopolymers intended for scaffold fabrication. Its unique physical, chemical, mechanical, and biological properties offer some important advantages over synthetic polymer materials. This review presents a critical overview of micro- and nanoscale cellulose-based materials used for scaffold preparation. It also analyses the relationship between the method of fabrication and properties of the fabricated scaffold. The review concludes with future potential research on cellulose micro- and nano-based scaffolds. The review provides an up-to-date summary of the status and future prospective applications of micro- and nanocellulose-based scaffolds for tissue engineering.

## 1. Introduction

Biopolymers are naturally sourced and have been reported to have good biocompatibility with the human system. Biopolymers have been proposed as a replacement for synthetic polymers used in tissue repair because of their nontoxic nature. Tissue engineering is an arm of regenerative medicine and for the most part makes use of scaffolds for the repair of tissues. Scaffold development with biopolymers for tissue repair has been at the forefront of material research, and cellulose, being the most abundant biopolymer, has been intensively investigated for this purpose.

Cellulose has been reported as the most abundant, naturally sourced biopolymer, with 150 billion tons produced yearly [1]. Plants mainly consist of cellulose, which provides them with their structural integrity. The use of naturally occurring polysaccharides for industrial applications has drawn the attention of many researchers. Natural polymers such as cellulose, starch, chitin, etc. have been used as the major materials for biomedical and packaging applications [2]. As a natural polymer, cellulose has been reported to be environmentally friendly, renewable, biodegradable, biocompatible, nontoxic, and covalently attach to many bioactive molecules [3]. As a result, it has been widely used in many medical and non-medical applications [4].

Microcellulose fibres have an area network of bundled microfibrils, which form slender and nearly endless rods. These microfibrils, when dissolved in strong acids, break down into short crystalline rods with a diameter range of 9–16 μm, known as cellulose microcrystals [5,6]. Cellulose microfibrils are usually longer than microcrystals. Cellulose microfibrils range from a few hundred nanometres for wood or cotton cellulose to a few microns for cellulose from animal sources such as tunicates [7].

Nanocellulose has a larger surface area, and this is responsible for its good reinforcement ability [3]. Micro- and nanofibres in cellulose polymer are the structural unit and consist of multi-dozens of chains that lie against each other, forming one straight chain called a cellulose fibril. Most of the fibrils have cellulose chains in a precise 3D order. Thus, there are two main forms: crystalline and non-crystalline (amorphous) [8]. Several forms of cellulose have been used in the fabrication of biomedical scaffolds such as powders [9], hydrogels [10], aerogels [11], and membranes [12]. Cellulose materials have been majorly used in scaffold fabrication with poly-lactic acid (PLA), polybutylene succinate (PBS), chitosan, and starch. Scaffold materials have been researched with binary and ternary blends of nanocellulose with other polymers with good mechanical strength [13].

At present, several reviews have reported on the use of biopolymers in biomedical applications [14]. Athukoralalage et al. (2019) reviewed the properties of nano-cellulosic materials and their use in 3D bio-printed hydrogels. The review discussion was focused on previous work on hydrogel 3D printing technology. Another recent review reported on [15] bacteria cellulose-based scaffolds for biomedical applications. The major review discussion was centred on bacterial cellulose, which differs from plant-based cellulose, as discussed in this study. Furthermore, Hickey and Pelling [16] reported on the general overview of cellulose source, types, and application in tissue engineering without describing the technique of scaffold fabrication. Other previous publications [17,18,19] have mainly focused on the application of scaffolds without comparing the fabrication technique with the types of scaffolds.

A number of publications in the literature have focused on the use of bacteria cellulose than plant cellulose. At present, the use of plant cellulose compared to microbial cellulose is gaining more attention. This is as a result of the recent revolutions of fabrication techniques of micro- and nanoparticles and recent toxicity studies. This review presents recent updates of micro- and nanocellulose-based materials in scaffold development for tissue engineering applications. It gives a critical review and analysis of previous research work in scaffold fabrication techniques with micro- and nanocellulose. Detail study of the chronological development and deficiencies of scaffold evolution is reported along with the highlights of the prospective applications, and further research on micro- and nanocellulose-based scaffolds are recommended in this study.

## 2. Plant Fibre Materials

Cellulose can be isolated from plant or animal sources and is composed of repeating units of glucose. Plant fibres are defined as natural elongated materials isolated from plant cell walls that have a specific tubular composite structure with a stack of piles. Each pile consists of primary and secondary walls [20]. The chemical composition of plant fibre is dependent on the source of fibre [21]. Common sources of plant fibres are cotton, sisal, and hemp [21]. Plant cellulose exists in combination with other components, mainly hemicellulose and lignin [22]. Therefore, during the isolation of cellulose, these compounds must be removed or at least eliminated. Lignin is bonded with hemicellulose and cellulose by ester linkages and hydrogen bonds. Lignin present in the cell walls of cellulosic fibres has been essentially responsible for the structural integrity of these fibres together with cellulose. Thus, natural or vegetable fibres in most cases (except for cotton) have been termed as lingo-cellulosic fibres [23]. The complex structure of lignin linkages with hemicellulose forms a matrix around cellulose molecules and results in it being very tough and difficult to break the network. The connection between the sugar monomer formed long chains and the interaction between these chains generates strong intermolecular forces between them. The high linearity of the cellulose molecule also results in a tightly packed nature of cellulosic fibres [24]. The polymeric structure and linkages of the polysaccharide resulted in the fibre’s strong intermolecular forces between its chains. The high linearity of the cellulose molecule accounted for the crystalline nature of cellulosic fibres [24].

Many procedures have been developed to produce cellulose fibre, for example, the preparation of spinnable solutions from cellulose using different solvents and conditions [25]. Viscose, modal, or lyocell cellulosic fibres can be generated with different physical properties such as the degree of crystallinity, molecular orientation, and chemical properties (adsorption of water and dyes) [26]. These fibres are usually produced with a significant number of inorganic additives, which are embedded inside the cellulosic fibres. The embedding of inorganic particles is essential to the creation of new fibres with different functional properties. The embedded inorganic particles act as the carrier of the functional properties. Cellulosic fibres are extensively used in many applications such as the manufacture of different types of apparel due to its comfort and ability to absorb moisture, making it comfortable against the skin [27]. In most cases, cellulosic fibres have been extensively blended with other polymeric fibres such as polyester and spandex, especially in the manufacturing of shirting.

### 2.1. Cellulose Fibre Architecture

Cellulose is a natural polysaccharide and consists of repeating units of monomer glucose connected through (1,4) beta D linkages. Plant biomass is composed of cells surrounded by primary and secondary cell walls. Cellulose and hemicelluloses are the main components of plant cell walls and form 34–75% of the primary cell wall and 50–80% of the secondary cell wall [28]. The aggregation of many cellulose molecules form fibrils. The fibril bundles combine to form cellulose fibres. The fibrils have both crystalline (ordered) and amorphous (disordered) regions that are the main reinforcement segment for plants, trees, algae, some marine creatures, and bacteria [29]. Bacterial cellulose is a type of cellulose produced by certain bacteria such as *Acetobacter xylinum*. *Acetobacter xylinum* is an extracellular product and a unique form of cellulose produced in nature. It is very pure cellulose with a narrow size distribution and high crystallinity [30]. Table 1 presents the main differences between plant-based cellulose and bacterial cellulose.

**Figure 1:**
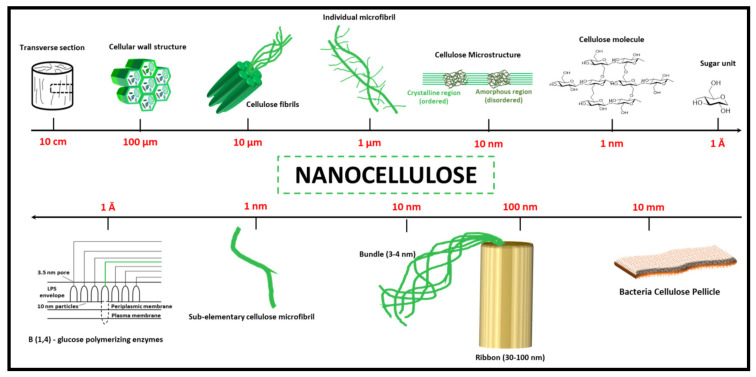
The hierarchical structure of cellulose. The upper part: plant cellulose; the lower part: bacterial cellulose. Adapted from [35]. Copyright, 2014, Elsevier.

The purity of bacterial cellulose is higher than that of plant-based cellulose. However, bacteria cellulose and plant cellulose have similar crystallinity, water retention capability, tensile strength, and biocompatibility. These characteristics of isolated cellulose are dependent on the source and method of preparation [35]. Figure 1 presents the hierarchical structure of cellulose, cellulose nanocrystals, and micro/nanofibrillated cellulose (the upper part of the figure), while the lower part presents the structure of bacterial cellulose.

Cellulose macromolecules are assembled in the semi-crystalline filament. The macromolecules consist of microfibres and nanofibres. The three-carbon monomeric units in the cellulose chain are bonded to the hydroxyl groups [36]. The hydrogen bonds play an important role in the structure of cellulose and the physicochemical properties of the molecule. The bond between a group of cellulose chains leads to the formation of micro-fibrils, which are jointly grouped to form typical cellulose fibres. Two different ends of the microfibrils in each cellulose polymeric chain possess carbonyl functionality and a supplementary hydroxyl group in carbon number 4. Stiffening of the cellulose chain has been a direct result of the banned free rotation of the cellulose rings by hydrogen bonds along with their linked-glycosidic bonds. However, the interactions of the van der Waals and hydrogen bonds lead to the formation of either crystalline ordered regions or amorphous disordered regions of the unique structure of cellulose [37]. The crystalline form has been known as type I, or cellulose I, and consists of a mixture of alpha-cellulose, beta-cellulose, triclinical structure, and monoclinical structure. Cellulose II is irreversible thermodynamically and more stable than cellulose I. Cellulose II can be prepared by treating the polymer with a concentrated alkaline solution such as sodium hydroxide. Different treatments of cellulose will lead to the generation of other crystal polymorphs such as cellulose III, IIIII, IVI, and IV [38].

The term microcellulose describe all forms of cellulose materials with diameters in the micrometre range, typically from 1 μm to 1000 μm, while nanocellulose describes the forms of cellulose-containing dimensions in the nanoscale (1–100 nm) [39]. However, both micro- and nanocrystalline cellulose can be produced by the extraction of crystalline regions from native cellulose using acid hydrolysis of disordered/amorphous regions, which link together assemblies of microfibrils [40]. Depending on the conditions of extraction, the crystalline region of the microcellulose can significantly vary in size and aspect ratio. This usually results in different types of fibrils, crystalline, and particle sizes (which may be in micro- or nano-size). However, they are normally anisometric [39].

### 2.2. Microcellulose Material Forms

**Figure 2:**
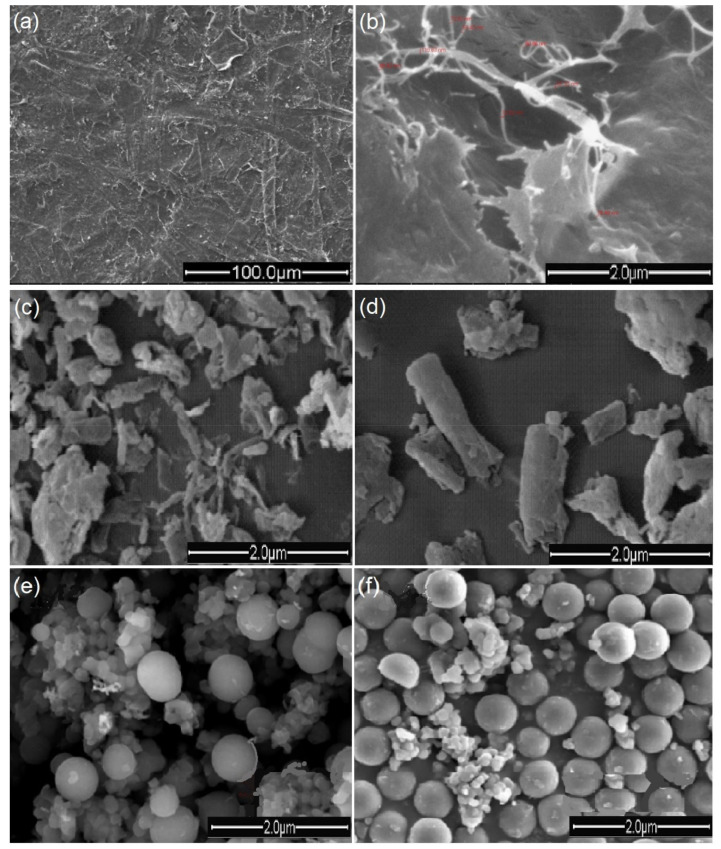
Scanning electron microscope (SEM) images of different forms of microcellulose materials. (**a**,**b**) Surface of micro-fibrillated cellulose from the bleached cotton stalk [49], (**c**,**d**) the surface of micro-microcrystalline cellulose from the cotton stalk [50], (**e**,**f**) microcellulose particles of cotton, adapted [51].

The micro-sized form of cellulose has two basic forms: micro fibrillated cellulose (MFC) and microcrystalline cellulose (MCC). The definition of these forms is based on the features in the physical and microstructural view of the material. These views differentiate the properties of each form and their effect when used as reinforcement in materials. Figure 2 shows the scanning electron microscope (SEM) images of the MFC and MCC forms. Cellulose microfibrils are composed of 18 or 24 cellulose chains with a variety of cross-sectional shapes, which are the original structure present in the natural plant [41]. MFC is produced from lignocellulosic biomass by mechanical treatment. The size distribution of MFC is wide, even if some fibres have diameters in the nanoscale. Microcrystalline cellulose (MCC) has been described as a purified, partially depolymerized cellulose. It has been sourced and isolated from woody and non-woody lignocellulosic materials and purified cotton linters [42].

It has been reported that cellulose microfibrils vary in size from 0.2 to 374 µm. The micro-fibrillated cellulose chain aggregates together to form macro-fibrillated chains [43]. However, microcrystalline cellulose has been reported as the non-amorphous region of the chain, which is divided into a well-organized crystal-surface and poorly organised crystal interior [44]. However, the amorphous or non-crystalline region has been attributed to mechanical damage or wood pulping treatment rather than the state of native cellulose [42]. Micro-fibrillated cellulose is a micro-size fibrillated unit obtained through a fibrillation process of cellulose fibres using mechanical shearing with or without enzymatic or chemical pre-treatment [45]. The small diameter of 3D fibrils allowed them to form a network of microfibres with an extremely large surface area. Micro-fibrillated cellulose has the typical structure of cellulose with crystalline and amorphous regions. It possesses a high viscosity and yield stress. It is sheer thinning and has a high water-holding capacity. The size distribution of these fibres are wide, their length varies from 50 to more than 900 μm, and the width from nano-size in some cases to micro-size [46]. Nie et al. [47] isolated micro-fibrillated cellulose from *Artemisia Vulgaris* Bast, using alkali degumming and TEMPO (2,2, 6,6-tetramethylpiperidine-1-oxyl) chemical treatment. The authors reported an average length of 850.6 μm and a diameter of 14.4 μm. Micro-fibrillated cellulose is often used in many industrial applications including biomedical and scaffold fabrication [48].

MCC is often prepared by the acid hydrolysis of cellulose at high temperatures. The reactive amorphous regions of the cellulose are selectively hydrolysed, which releases the crystallites out of the chains. The results of the acid hydrolysis of the aqueous suspension have often been referred to as microcrystalline cellulose suspensions [23]. The particle size of isolated microcellulose materials is dependent on the cellulose source, pre-treatment operation, and the preparation process. Recently, Nie et al. [47] used an alkali degumming process to prepare micro fibrillated cellulose and a significantly small particle size was obtained, compared to the result of Tamaddon and Hosseinzadeh [5]. Furthermore, microcrystalline cellulose has been prepared with different techniques. Table 2 shows the comparison between the obtained particle size and preparation technique.

Modification of hydrolysis conditions such as temperature, time, and acid concentration has been reported to have a significant impact on the degree of polymerisation of MCC. However, hydrolysis conditions have very little impact on the degree of crystallinity of MCC. Additionally, the moisture within the pores of MCC has a significant effect on its physical and chemical properties. It also has a significant impact on its internal lubricant, which in turn affected its tensile strength, compaction, and even viscoelastic properties. The heat stability and relatively constant viscosity at different temperatures of MCC suspensions have been established from previous research. Its excellent mouth-feel properties have also been explored in the pharmaceutical and food industries to extend starches, control ice crystal formation, the binder in pharmaceutical tablets, confections, stabilise foams, and cosmetics [55]. Table 3 presents the progressive chronological studies of microcellulose-based materials.

### 2.3. Nanocellulose Material Forms

**Figure 3:**
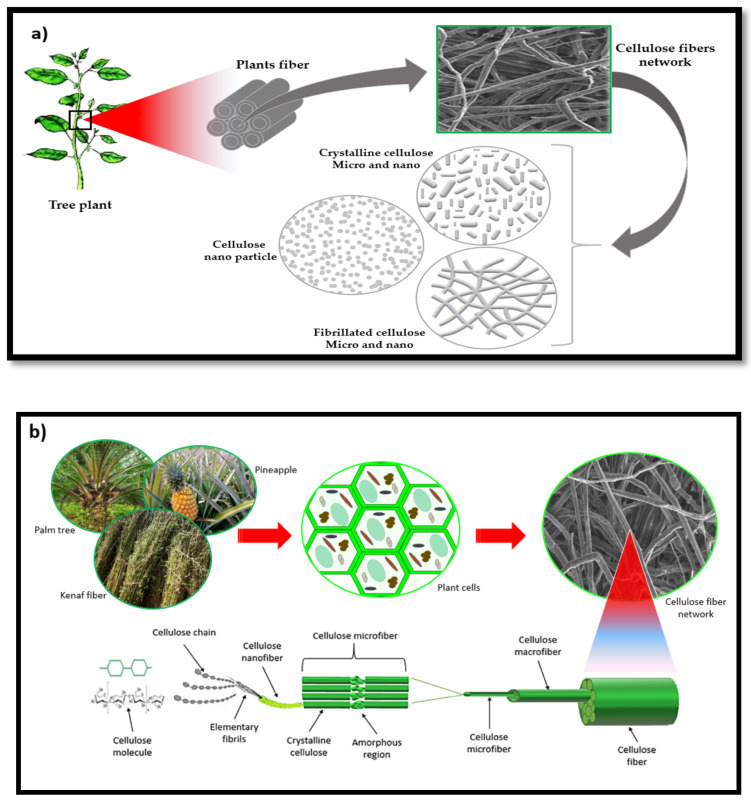
(**a**) Classification of nanocellulose materials based on microstructural forms. (**b**) Schematic diagram of different scientific forms and structure of cellulose fibre.

Nanocellulose materials have been classified into two main classes. Cellulose nano-objects, which include materials with one, two, or even three external dimensions in the nanoscale (1–100 nm), and nanostructured cellulose materials with an internal composition of inter-related constituent parts in which one or more of these parts is in the nanoscale. Cellulose nano objects include cellulose nanocrystals, cellulose nanofibrils (CNFs), and cellulose nanoparticles. At the same time, nanostructured cellulose materials include cellulose microfibrils, micro-fibrillated cellulose, and bacterial cellulose [40]. Nanocellulose is often used by researchers to enhance the strength of biomaterials. Nanocellulose possesses high mechanical strength and environmental sustainability [72]. Many researchers have categorised nanocellulose materials into three main types (Figure 3): nanofibrillated cellulose, nanocrystals, and nanoparticles [73]. These three types have a relatively similar chemical composition, but different degrees of crystallinity, particle size, and morphological properties [3].

Soyekwo et al. [74] describes nanofibrillated cellulose (NFC) as long flexible nano-size fibres, 1–100 nm in diameter and 500–2000 nm in length, and chemically composed of 100% cellulose with both crystalline and amorphous regions. The average size of NFC particles varies and depends on the method of preparation. Recently, NFC has been economically produced from various cellulose sources such as pulp and cotton via a series of surface modifications to enhance their performance in the desired application [75]. A single nanofibre has outstanding stiffness as well as good chemical and thermal stability. Over the last decade, NFC use in scaffold fabrication has gained significant interest, and this is due to their low coefficient of thermal expansion, high Young’s modulus, high strength, biocompatibility, low weight, and renewability [76]. The NFC dispersed solution and hydrogel precursor have been cross-linked with a relatively facile and mild method for hydrogel scaffold for tissue engineering [77]. At present, tissue scaffolds using a NFC aerogel have been widely fabricated [3].

**Figure 4:**
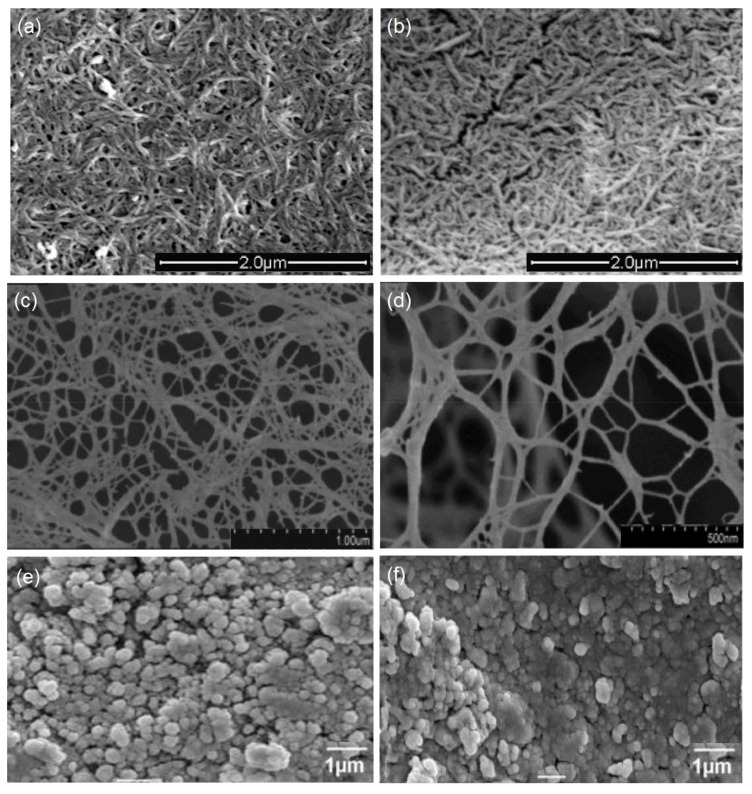
Scanning electron microscope (SEM) images of different forms of nanocellulose materials. (**a**,**b**) Nanocrystalline cellulose obtained from cotton and cotton stalk pulps, adapted from [83]. (**c**,**d**) Surface of nanofibrillated cellulose from cotton, adapted from [84]. (**e**,**f**) The surface of cellulose nanoparticles from cotton, adapted from [85].

Nanocrystalline cellulose (NCC) has been extracted from cellulose fibres by acid hydrolysis and characterised with high strength [78]. NCC has otherwise been called cellulose nano-whiskers or cellulose nanocrystals [79]. Isolated NCC has been described as shorter and has a rod-like shape compared to NFC. Figure 4 shows the SEM images of the nanocellulose materials. The acid hydrolysis method used by [76] was to reduce the average diameter using an ultra-turrax and micro-fluidizer. A similar diameter has been obtained by [80]. The diameter of NFC is diverse based on the preparation process, as presented in Table 4. In [81], the authors obtained NFC with a small diameter of 15 nm by using the blending method, which was smaller than the one obtained with the electrospinning process (70–130 nm) by [82].

Over the past few years, NCC has been investigated for many medical applications including tissue-engineering scaffolds. Its biocompatibility and other properties that are similar to NFC make it suitable for in vitro and in vivo usage. Shaheen et al. [91] fabricated a NCC with a high stiffness similar to titanium, copper, and bronze materials used in bone implants. Furthermore, the high aspect ratio, surface area, and crystallinity as well as low elongation at break, in turn, support its strength for 3D scaffolds as a load-bearing material [92].

Cellulosic nanoparticles (CNPs) are another cellulose-based nano-sized short structure material of cellulose. Gu et al. [93] characterised CNPs and described them as short needle or ribbon-like particles. Furthermore, their properties are dependent on the source of the cellulose, and it appears as a rod or spherical shape. CNPs have been prepared by various hydrolysis methods, the removal of the amorphous regions of cellulose, and dispersal of the crystalline region [94]. Table 5 presents the progress of studies among the nanocellulose-based materials.

## 3. Cellulose-Based Scaffolds in Biomedical Engineering

Tissue-engineering technology has advanced with the potential to grow desired cells, tissues, or even whole organs. Regeneration of damaged cells, tissues, or organs of the human body with natural and synthetic polymers has been achieved to a great extent [108]. The role of natural polymers such as cellulose as a replacement of synthetic polymers has been on the increase in biomedical and tissue engineering applications [109]. Langer and Vacanti in the 1990s were the first to describe and define the term “tissue engineering” and its effect of materials on the cells [110]. Since then, many publications and research have been undertaken to fabricate three-dimensional structured scaffolds for cells to adhere and proliferate. The advancement in tissue engineering has created an avenue for a new era in regenerative medicine. The discovery of rapid prototyping for part production has also enhanced the fabrication of complex scaffolds. Micro- and nanocellulose-based materials and hybrids have been used as bio-ink for 3D printing, which provides a unique methodology for the customisation of desirable biomedical scaffolds [106]. 3D scaffolds with controlled pore structure and orientation, shape, and size have been fabricated from different materials for the regeneration of a variety of cells such as bone regeneration [111], muscle regeneration [112], ligament regeneration [113], and tendon regeneration [114].

### 3.1. Development of Cellulose-Based Scaffolding

Cellulose-based materials were among the first developed materials for scaffold forming due to cellulose immune response (biocompatibility) and better overall interactions with various cell types compared to many synthetic polymers [115]. However, a key need for effective tissue engineering is to provide the cell’s growth and proliferate requirements, which is the main role of the scaffolds [116]. The purpose of scaffolds, as reported by Berthiaume et al. [116], is their ability to provide a suitable environment for the attachment and migration of cells, biochemical retention, diffusion of both nutrients towards the cells, and mechanical support. Among the natural polymers often used in tissue scaffold fabrication are proteins (collagen and fibrin) and polysaccharides (cellulose and chitosan). Several reports on cellulose-based scaffolds have reported biocompatibility and less or no cytotoxicity. However, there remain issues with potential immunogenicity in some types of tissues.

### 3.2. Method of Preparation of Micro- and Nanocellulose-Based Scaffolds

**Figure 5:**
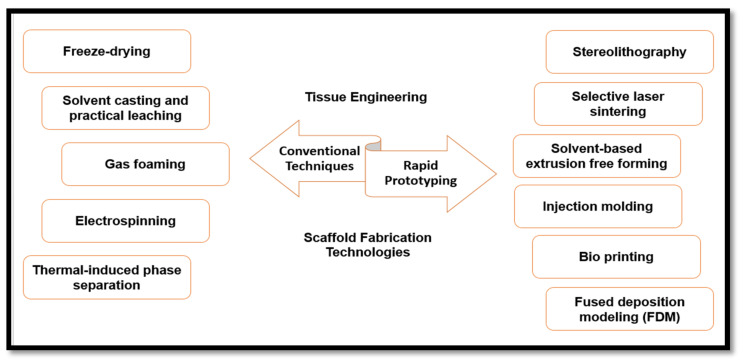
Types and classification of scaffold fabrication technologies of micro- and nanocellulose-based materials.

Fabrication of scaffolds has been described as a major area of tissue engineering, regenerative medicine, and biomedical research [117]. Previous research work in this area has been focused on restoring, improving, or maintaining the functionality of the growing cells. Scaffolds have been used in tissue engineering technology to provide a multi-layered matrices nested network for growing cells, which is required for growth, proliferation, and cell differentiation [118]. Scaffolds have also been utilised to facilitate the repair of damaged and defective tissues such as skin during burn injuries. Furthermore, fabricated scaffolds for the growth of whole organs have also been proposed. Many organs have been suggested for growing in scaffolds such as kidneys or livers with 3D printing technology. With a view to achieving good mechanical support in tissue repair, in vitro growing, and proliferation of cells, many fabrication techniques have been designed, as summarised in Figure 5. Eltom et al. [118] classified the design of a functional scaffold into two parts or stages: the micro-scale level, also called the cell level, and the macro scale, also known as the tissue level. The cell level design involved consideration of the scaffolding functionality in the environment of its use and function of cells. The macro-scale level or tissue level involves tissue construction in maintaining the adequate transportation of nutrients and possesses the mechanical properties of multicellular tissue.

#### 3.2.1. Conventional Techniques for Micro- and Nanocellulose-Based Scaffold Fabrication

Several researchers have developed novel techniques for the fabrication of different shapes of 3D polymeric scaffolds [115]. A significant number of scaffolds have been designed and fabricated with the techniques of that era, known as conventional techniques for scaffold fabrication. These techniques were done with consideration of the bulk, surface properties of the materials and the proposed function of the scaffold. Freeze-drying has been one of the most widely used traditional techniques in scaffold fabrication for biomedical applications. The technique uses water and ice crystals instead of organic solvents, and it does not require high temperatures. Thus, the pore size of the resulting scaffolds is manageable, but it is still not possible to obtain homogenous pores. However, the authors in [119] reported some drawbacks in this technique such as energy consumption and irregular sized pores. Table 6 (Part A) shows a summary of conventional techniques used in the scaffold fabrication of micro- and nanocellulose-based material.

Solvent casting and particle leaching have also been used as another traditional technique in scaffolding. Kanimozhi et al. [131] compared freeze drying and salt leaching techniques in the fabrication of a composite cellulose scaffold. The result revealed that salt-leached scaffolds were easier to form into desired shapes. Furthermore, the results of the biocompatibility experiment showed that the salt leaching scaffold had better cell viability than the freeze-dried scaffold. Eltom et al. [118] described the salt leaching technique as the dissolution of the polymer in different solvents and salt particles. However, scaffolds produced by the salt leaching technique cannot guarantee the interconnectivity of pores due to the adjacent salt particles that are in contact with each other. The salt contact results in the inconsistent macrostructure and microstructure of the prepared scaffolds. Chen et al. [132] enhanced this method by combining particle leaching and freeze-drying techniques and by using pre-prepared ice particulates as the particle material. The authors reported that their scaffold possessed good mechanical strength handling, and had no effect on the cells’ proliferation. This method has been used because of its low-cost and ability to build a highly porous 3D scaffold. Zubairi [133] studied the residual effect of sodium chloride on cell growth media and revealed that residual sodium chloride might be considered as too small to produce any adverse effect on cell growth. A bacterial cellulose scaffold was prepared using this technique, and field emission scanning electron microscopic analysis revealed a porous structure of the scaffold. It also exhibited a very high swelling ratio, indicating enhanced water absorption and nutrient exchange capacity. Moreover, an in vitro biocompatibility study indicated good cell adhesion, penetration, and proliferation [134].

The gas foaming technique makes use of high temperatures and organic solvents to dissolve the polymer. Inert gases are also used to pressurize the obtained polymer melt with water or fluoroform until saturated full gas bubbles have been formed [135]. The control of the pore size and interconnectivity in gas foaming is done by the variation of process parameters such as temperature, CO_2_ pressure, depressurization rate, and particle size of salt [136]. These parameters have a significant effect on the physical and mechanical properties of the cellulose scaffold. A pore size within the range of 116 to 418 μm has been generated using this fabrication technique, and the porosity ranged between 69.8 and 92.3% [136].

The electrospinning method involves the use of high voltage electricity for scaffold fabrication. It has been reported by Li et al. [137] as a very complicated technique for the fabrication of nano-fibrous scaffolds. He et al. [138] used this method to fabricate cellulose nanocrystal based scaffolds and evaluated its potential use in tissue engineering. The scaffold exhibited good physical properties and mechanical properties. The technique was essentially nontoxic to human cells and enhanced rapid proliferation not only on the surface, but also deep inside the cellulose layers. However, many drawbacks have been reported regarding electrospinning. The drawbacks include the use of toxic solvents, low rigidity, difficulty in controlling pore size and pore shape, dependency on many variables and the densely packed fibres in two-dimensional (2-D) array, which may impede their applicability in tissue regeneration. [139].

**Figure 6:**
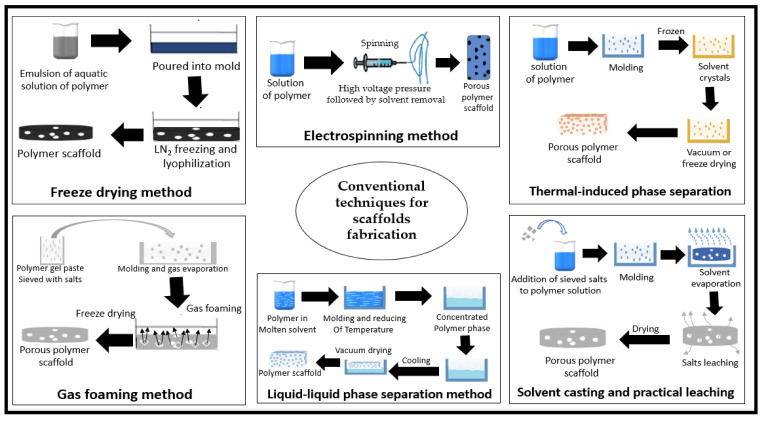
Schematic drawing of the biopolymer forming process of conventional techniques for scaffold fabrication.

Thermal-induced phase separation is a different method that involves alteration of temperature in forcing phase separation of the polymer solution. The solvent is eliminated with freeze or vacuum drying and this results in a porous, nanoscale fibrous network. This method has been used for the fabrication of many thermoplastic and polymeric based scaffolds [140]. Organic solvent residues in the scaffold are the main disadvantage of this method, which has been reported to affect the viability and proliferation of growing cells [124] The mechanical properties of the foams could be tuned by changing the overall porosity and the hard segment content of the polymer. Figure 6 shows a summary of the conventional techniques used in scaffold fabrication.

#### 3.2.2. Rapid Prototyping Techniques for Scaffold Fabrication

Rapid prototyping techniques are manufacturing methods that make use of computer-aided design without the need for any tools or equipment. Rapid prototyping has often been referred to as solid free-form fabrication [141]. Table 7 (Part B) shows the summary and schematic drawing of the different rapid prototyping techniques.

Stereolithography is one of the rapid prototyping techniques used to manufacture solid 3D scaffolds. Stereolithography involves consecutively printing a layer-by-layer of photosensitive liquid resin with an ultraviolet laser until a 3D scaffold is fabricated [118]. Cellulose nanofibre (CNFs) composite scaffolds have been fabricated using this technique with high and tunable compressive modulus and high porosity of approximately 90%. The authors reported that CNFs in the composite scaffolds played a significant role in structural shape integrity, porous structure, and mechanical strength. Additionally, the cytotoxicity experiment revealed that cells had good differentiation and viability [125]. Another rapid prototyping technique is known as selective laser sintering, like stereolithography. However, this method uses laser light instead of UV to sinter powdered material in thin layers. Selective laser sintering has been utilised to fabricate scaffolds from various polymeric materials including cellulose-based materials with excellent user control over the microstructures of the manufactured scaffolds [142]. In a study reported by Shuai et al. [143], the authors prepared a cellulose nanocrystal-based scaffold using the selective laser sintering technique. The scaffold was reported to possess good physical and mechanical properties with excellent capability of supporting cell adhesion, proliferation, and differentiation. The solvent-based extrusion free-forming technique is a powder-based rapid prototyping method, the principle of which has been the realisation of liquid to solid transition through solvent evaporation. This can done in the presence of a binder, which enables the incorporation of cells during the scaffold printing process [144,145]. Lu et al. [146] studied the factors affecting the structure of the scaffold with this technique and concluded that the solvent content of paste is the most important parameter that determines the filament’s ability to span and retain the planned height [146]. Limited studies about using this technique in tissue engineering scaffold fabrication due to the effect of solvents on the growing cells makes the solvent-based extrusion free-forming technique less used in this field.

In 3D printing, 3D objects from designed computer models have been produced. The adhesive was printed with a selectively desired powdered material yielding a complex 3D object from the desired powder material [147,148]. This method was improved by Xu et al. [149] in terms of stability and fidelity by cross-linking the polymer with other auxiliary materials, which showed prospects in terms of skin repair and wound healing. Printing of biomaterial using 3D printers utilised in the fabrication of 3D scaffolds from different biomaterials with solvent-free, aqueous-based systems Bio 3D printing has been classified into cellular and acellular printing [150]. Acellular bio 3D printing has been used in the manufacture of scaffolds alone without involving target cells, resulting in higher accurate scaffold dimensions with greater shape complexity. In contrast, cellular bio 3D printing involves the integration of the desired cells and other required growth agents with the biomaterials during the printing process, resulting in a high speed of printing process [135].

**Figure 7:**
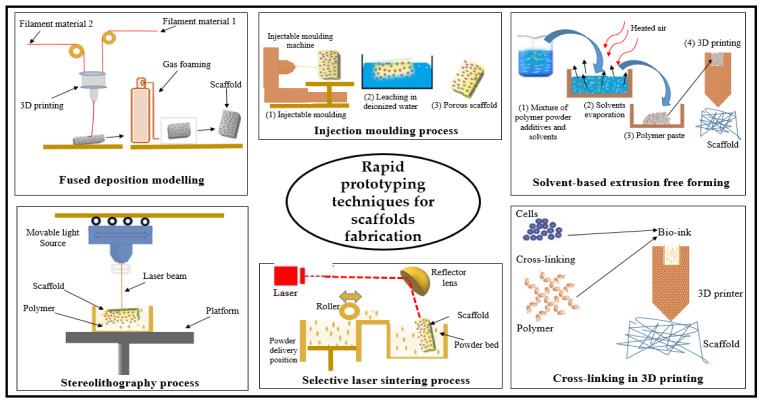
Schematic drawing of biopolymer forming rapid prototyping techniques for scaffolds.

Fused deposition modelling is another recent technique in scaffold fabrication, which uses computer-controlled extrusion of desired materials to create 3D multiple layer scaffolds. By using this technique, it is possible to obtain cellulose-based scaffolds with 70% porosity and a fully interconnected network of pores, and the porosity could be increased by altering the parameters. The authors reported pore sizes ranging from 200 to 500 μm, with excellent mechanical properties. Biocompatibility experiments have shown that cells were viable and that there were no signs of cellular death even after three weeks of culture [151]. The technique has limitations in its application to biopolymers; however, it has been using a low temperature and can be used in designing scaffolds under the different aspects of fabrication [118]. Figure 7 shows a summary and schematic drawing of the different rapid prototyping techniques.

The biggest advantage of rapid prototyping techniques over conventional preparation techniques is the ability to individualise application needs to efficiently apply scaffolds. Furthermore, using rapid prototyping, the scaffold shape can be controlled as well as the inner pore structure with high reproducibility. Table 7 highlighted the difference between rapid prototyping and conventional preparation techniques.

### 3.3. Properties of Micro- and Nanocellulose-Based Blend Scaffolds

Cellulosic material has good physiological inertness and biocompatibility, and these properties have attracted its use in tissue engineering [3]. Both micro- and nanocellulose materials have been used in the fabrication of scaffolds with various techniques, and this can be divided into three main aspects: (i) cellulose membrane scaffolds, (ii) cellulose matrix composite scaffolds, and (iii) surface modified scaffolds.

#### 3.3.1. Physical Properties

The natural composition of cellulose-based materials has provided a suitable cell-friendly environment for growing cells in tissue engineering scaffolds and encourages cell attachment proliferation. Low cytotoxicity and high porosity of cellulose-based scaffolds have made these types of scaffolds the favourable choice for culturing a variety of cell types. ME Alemán-Domínguez et al. [129] prepared bio-scaffolds from a microcrystalline cellulose binary blend with the 3D printing technique to culture sheep bone marrow cells. It was reported that their scaffold had 450–500 µm pore size and 50–60% porosity. The study also recorded a significant enhancement of cell proliferation. The researchers concluded that these materials had suitable properties for tissue engineering scaffolds. Salmoria et al. [126] used the selective laser sintering technique to fabricate starch–cellulose and cellulose–acetate scaffolds. The result revealed that specimens with smaller particle size had a higher degree of sintering, mechanical strength, and a significant level of closed pores. This was indicated by the density measurements and fractography analyses. Cellulose nanocrystals, on the other hand, had a significantly smaller size (5–6 nm) than microcrystalline cellulose (20–24 μm) and starch. The cellulose nanocrystal based hydrogel scaffold by Yin et al. [157] showed a smaller pore diameter than the microcrystalline cellulose (about 80–120 µm). The authors reported that CNC significantly enhanced the physical property of the hydrogels, and played a vital role according to the rheology and swelling results. Similarly, He et al. [138] fabricated cellulose nanocrystals using the electrospinning technique. It was reported that the addition of CNC increased the tensile strength and elastic modulus. The thermal stability of the scaffold was also significantly improved. Other studies have concluded that using nanocellulose based scaffolds have better cell attachment due to the enhancement of physical and mechanical properties, which mainly affect the cell–scaffold interaction [158,159]. Novel polymer ternary blend scaffolds have been fabricated by Ninan et al. [160] to culture fibroblast cells. In their research, it was reported that the optimised ternary blend scaffold was highly porous (up to 88%) and with significantly reduced pore sizes. The viability of cultured cells reported in the study was high. The ternary blend scaffold possessed high biocompatibility and low cytotoxicity. The attachment of cells to the scaffold surface has been an important feature of concern. However, cellulose-based materials tend to exhibit good cell attachment, which is enhanced by surface modifications to the scaffold. Further cell attachment properties have been undertaken with the addition of peptide coatings, protein, or plasma treatment [161].

#### 3.3.2. Mechanical Properties

According to Pelling and Hickey [16], the mechanical properties of cellulose-based scaffolds depend mainly on its chemical structure, the crystal structure of the materials, and their degree of crystallisation. Ideally, tissue engineering scaffolds should have good mechanical properties in terms of the necessary strength to allow surgical handling in implantation. Atila et al. [162] used hydrophilic pullulan to enhance the mechanical strength of the cellulose–acetate scaffold. The addition of pullulan significantly increased fibre diameter, thickness, and porosity of the cellulose scaffold. Many materials have been produced with good mechanical properties, but due to the high porosity needed for in vitro, have failed when implanted due to insufficient capacity for vascularisation. The particle size of the precursor materials play a critical role in the mechanical properties of the prepared scaffold, and smaller particles size provide higher mechanical properties to the scaffold. Therefore, most current researchers have shown a preference for the use of nanocellulose for scaffolding [126]. The mechanical properties of cellulose-based scaffolds can be significantly enhanced with microspheres [163] as the microparticles improve the interconnectivity of the shaped pores. The improved connectivity leads to better nutrient supply and fluid exchange when the scaffold is in use. The microparticle sizes also have a significant impact on the mechanical properties of the formed scaffolds. Novel research, which would account for a balance between the mechanical properties and porous architecture sufficient to allow cell infiltration and vascularization, has not been fully explored.

#### 3.3.3. Biodegradability

The lack of cellulose degradable enzymes such as cellulase make the material non-biodegradable in vivo. However, the form and chemical derivatisation of crystalline cellulose and blending of cellulose with other polymers or materials may affect its degree of degradation and even immune response. In vitro degradation of oxidised cellulose materials comprises two main phases. The first phase is the initial rapid degradation, which leads to the degradation of approximately 70–80% of the cellulose sample. The second phase has a slower degradation of an additional 5–10% of the sample. This leaves a small amount of non-resorbable cellulose-based material [164]. With periodate oxidation, many recent types of research have attempted to enhance the biodegradability in vitro [165]. Various micro-organisms use cellulose as a carbon source; hence, they degrade it with specific enzymes, but not on an in vivo scale.

Further experiments on in vivo degradation showed the marked degradation of oxidised cellulose membranes at all-time points, with the most rapid degradation over the first 2–4 weeks [164]. Natural or spontaneous biodegradation of cellulose chains leads to a slow breakdown of unaltered cellulose on the in vivo scale. In vivo, non-enzymatic degradation of cellulose has not been adequately studied yet [166].

#### 3.3.4. Biocompatibility and Cytotoxicity

The term biocompatibility is described as the ability of a material to function in living tissue without complications. The biocompatibility of a material is often dependent on its cytotoxicity and the immunological response of that material on its exposure to the body fluids or cells [167]. A few pieces of research on the biocompatibility and cytotoxicity of the cellulose-based material have been conducted. Several studies have provided results on biocompatibility and cytotoxicity, but have not been fully explored due to a variety of cellulosic fibre sources, range of different methodologies, and sample preparations. A significant number of studies have confirmed that cellulose could be generally considered to be biocompatible with only moderate or no foreign body responses in vivo [168,169]. Ul-Islam et al. [158] analysed cell–scaffold interaction using a cancer cell line and cellulose/chitosan scaffolds. Strong adhesion and negligible aggregation of cancer cell lines were reported in the scaffold matrix and better cell–scaffold interaction than cell–cell interaction [158]. Ramphul et al. [170] reported that due to the presence of several OH groups, cellulose is more hydrophilic and hence will promote cellular interactions. The strong interaction between human cells and cellulose scaffolds have been linked with the downregulation of Notch receptors in the growing cells due to direct interaction between the strange material and the cells. Real-time polymerase chain reaction (RT-PCR) has been used to verify this hypothesis. Primers for notch −1 to 4 receptors have been used and revealed that the cell signalling pathway is responsible for the generation of the expected strong connection of cell lines with the scaffold and a weak connection among the cell lines themselves [158]. According to a study conducted, the interaction of NFC with the cells led to a high level of secreting inflammatory cytokine tumour necrosis factor-alpha (TNF-α), which could be the result of fibre-receptor interactions, which are highly dependent on the surface chemistry of the material [159]. The secretion of TNF-α could be inhibited by the introduction of surface charges on the surface of cellulose scaffolds, which have been done by the same authors [159]. Other studies have suggested that the human body can fully degrade cellulose with cellulolytic enzymes, which would have inevitably caused some incompatibility [171]. The toxicology of cellulose-based materials used in tissue engineering has been conducted on the viability, proliferation of cells, and cytotoxicity. Table 8 shows a summary of some recent research into the toxicology of cellulose-based materials. Generally, there has been no evidence for serious impact or damage of cellulose-based materials at both the cellular and genetic level as well as in vivo organ and animal experiments. However, the inhalation of intake of a high percentage of nanocellulose may induce pulmonary inflammation due to the easy self-aggregation and non-degradation of nanocellulose in the body of animals.

### 3.4. Classification of Micro/Nanocellulose-Based Scaffolds

Over the past decade, several micro- and nanocellulose-based materials have been extensively studied and investigated as biomedical materials for various applications including scaffold fabrication for tissue engineering. However, an ideal tissue engineering scaffold possesses many other properties apart from the biocompatibility to evade adverse tissue reactions. Additionally, excellent mechanical properties and an interconnected porous structure are needed with retained hollow spaces to proliferate the growing cells. A significant number of studies have recently proven that cellulose-based material scaffolds with both micro- and nanoscale structures are suitable for tissue engineering [30,184,185]. Cellulose-based biomaterials offer some important advantages over conventional synthetic materials and show great promise to advance the frontier of scientific knowledge. Physical mixtures of two or more polymers, referred to as polymer blends that could be the same polymer (homo-polymers) or different polymers (copolymers), have been of great technological significance [186]. Cellulose-based blends have been extensively investigated because of their theoretical and practical importance, particularly in scaffold fabrication.

#### 3.4.1. Micro- and Nanocellulose-Based Binary Blends Scaffold

Polymer binary-blends have been described as materials composed of two individual polymers to produce an optimised one for the target application. Cellulose binary blends offer the potential to combine the unique properties of cellulose with other polymers with desirable properties in tissue-engineering scaffolding. Cellulose and chitosan have been proposed as promising systems for creating new materials such as fibres, blended films, and sponges. However, the two polymers in the binary-blends must form inter- and intra-molecular interaction between their macromolecules, which is referred to as the compatibility of the polymers [187]. The similarity in the chemical structures of the polymers can be used to predict their compatibility. Polymers with similar chemical structures are likely to be compatible (homogenous) with each other and vice versa [72]. Binary-blends of materials could be obtained from either solid (under high pressure and shear deformation) or liquid states [188]. The fabrication process of these materials is done by the dissolution of the desired two polymers separately in the same common solvent, or by blending the solid powder of the polymers and then simultaneously dissolving both polymers in the solvent.

#### 3.4.2. Micro- and Nanocellulose-Based Ternary Blends Scaffold

The main purpose of blending the polymers is to obtain materials with additional properties with a minimum sacrifice of their original properties. The blending of three materials is referred to as ternary blends and have been a well-established route to achieving a certain number of physical polymer systems. It is often more rapid and economical than the development of new materials [189,190]. Ternary blends of micro- and nanocellulose-based materials for scaffold preparation have been researched. The material blends’ miscibility is often enhanced with a plasticiser, which is often organic. Ternary blend scaffold materials that are micro- to nanocellulose-based with polylactic acid, chitin, and starch are most common because of their compatibility with the human system [191]. These blends have been used for skin tissue repair. The blend ratio has been reported to have a significant effect on the morphology, surface area, crystallinity, thermal properties, miscibility, surface hydrophilicity, and cell culture. Furthermore, ternary blend scaffolds have also been fabricated for skin regeneration applications with gelatine, cellulose acetate, and elastin [192]. The use of micro- and nanocellulose in ternary or multicomponent biopolymer blends for scaffold fabrication has enhanced the functional properties of scaffolds.

## 4. The New Role of Cellulose-Based Scaffolds Bioengineering

Biopolymer-based materials have been increasingly in use for the production of medical devices, pharmaceutical products, and tissue engineering scaffolds [193]. The chemical and mechanical properties of cellulose-based scaffolds are resistant to chemical reactions and enhanced cell growth factors [194]. Several techniques have been developed to shape cellulose-based materials into complex frameworks and support it with many materials to enhance their properties. The enhancement of the properties in the cellulose-based scaffold is majorly due to the desired demands for the growth, proliferation, differentiation, and shaping of cells in the environment in vivo conditions [195]. Furthermore, scaffolds must play a major role in the 3D shaping of the growing cells, forming natural tissue and then a whole organ. 3D printing technology has allowed researchers to fabricate more complex structures from cellulose materials by integrating layer-by-layer slices of the designed and desired objects. This revolutionary technology has been used in the regeneration of many organs such as articular cartilages [196].

### 4.1. Issues and Potential

The use of nanoparticles has raised many concerns among researchers. F. Ghaemi et al. [197] evaluated the toxicity of NFC and concluded that lower cytotoxicity to cells might be gathered from the NFC in scaffolds. In contrast, Kim et al. [179], even though there was no irritation to the skin and eyes, reported significant cytotoxicity to skin cells. However, Roman [198] reviewed the literature on the cytotoxicity that may be gathered from cellulose-based materials where it was concluded that no toxicity was demonstrated upon dermal and oral administration. Although a huge number of studies has been done to fabricate different scaffolds with different techniques, several grey areas still exist. Novel research, which would account for a balance between the mechanical properties and porous architecture sufficient to allow cell infiltration and vascularization, have not been fully explored. Furthermore, research on the culturing of cells on scaffolds, the long-term toxicology, and potential mutagenesis have not been done. At present, the cytotoxicity tests reported are without its effect on the genetic properties of the cells. In vitro growth of a whole graft, organs have been yet another obstacle and some simple structured organs (ex, skin) have already been fabricated, but some of them possess inflammatory and allergic responses [199]. The use of cellulose-based materials (biopolymer) in scaffold fabrication has many advantages over synthetic polymer materials. Still, the long-term effects and potential health risks must be widely studied to avoid possible undesirable mutations. Several studies have provided results that have not been fully explored due to a variety of cellulosic fibre sources and the range of different methodologies for scaffold preparations. In vivo, non-enzymatic degradation of cellulose has not been adequately studied yet. Moreover, the innovation of new cellulose composite materials with micro- and nanoscale tissue engineering techniques has been one of the challenges (i.e., to create fully bio-mimetic artificial tissues). As the complexity of the application increases such as in highly dynamic tissues, an active remodelling of the scaffold design and fabrication would be required.

### 4.2. Future Prospect and Applications of Micro- and Nanocellulose-Based Scaffold

Cellulose-based scaffolds have been widely used in tissue engineering allocation because of their high ability to form a different three-dimensional porous structure that mimics biological tissue and has excellent mechanical properties [200]. Reports of scaffolds prepared from cellulose hydrogels [201] showed that injectable nanocellulose hydrogels have gained wide application due to their minimally invasive injection into the target sites and ability to match irregular defects. Xu et al. [202] utilised cellulose hydrogel as a template for a nerve regeneration study using polyaniline as the nerve guidance conduit. In vitro and in vivo studies were conducted using Sprague–Dawley rats, and the composite hydrogels exhibited good mechanical and biocompatibility properties. The biocompatibility of cellulose micro- and nano-structures and electrical conductivity of polyaniline induced the adhesion and allowed the extension of neurons. Further studies on this could result in the use of cellulose hydrogel in nerve regeneration. Recently, 3D printing technologies of cellulose-based hydrogels for the fabrication of 3D shapes has also received extensive interest in tissue engineering scaffolds [203]. Markstedt et al. [204] developed revolutionary biomimetic all-wood-based ink for the regeneration of human organs, which was formed from the crossing-link of a matrix reinforced by cellulose nanofibres. The ink used was the main material for the 3D printer, which had a high water content and is preferable in tissue engineering applications. Similarly, Wu et al. [205] used 3D printing technology to fabricate binary blending alginate/NCC hydrogel scaffolds to mimic the liver structure. The hydrogel scaffold had excellent mechanical properties where hepatoma and fibroblasts cells incorporated into the scaffold very well, and the cell mortality was minimal during printing [205]. Artificial skin from a cellulose-based material has been widely developed and become commercially available. It was reported that cellulose-based scaffolds support the adhesion and growth of dermal fibroblasts [206], and supported colonisation with fibroblasts to a lower extent than the adhesion, spread, and growth of keratinocytes [207]. Khan et al. [163] used confocal microscopy to study the penetration of keratinocytes into the scaffolds and reported that the penetration was up to 300 μm in depth. The same author did in vivo wound healing and skin regeneration experiments in mice and revealed complete skin regeneration within only two weeks, with much higher wound closure efficacy than that of the control. Another study was done by Huang et al. [208] that aimed to regenerate skin using cellulose-based scaffolds and adipose-derived mesenchymal stem cells. The artificial skin exhibited extremely high biological activity, a strong induction of cell recruitment, migration, growth, and upregulation of gene expression of relevant factors, resulting in excellent wound healing characteristics. The cell–scaffold interaction promoted the proliferation of cells and enhanced cell viability. With 3D technology, chemically modified NFC has been used as a bio-ink for printing and modifying film surfaces as artificial skin [209]. Bone and cartilage tissue replacements have also been fabricated using cellulose scaffolds. Cecen et al. [210] evaluated the biocompatibility, mechanical properties, and the interaction between the cellulose-based scaffold and chondrocyte-like cells. The results of the histological evaluation showed that cells produced an extracellular matrix. The extracellular matrix can be observed with the growth of proteoglycan and types I-II collagens. This growth showed that cellulose-based material promotes the proliferation of cartilage cells. The reverse templating method has been one of the most used and reliable methods in bone regeneration applications, which allows for mathematically defined and control pore geometries of the scaffolds, where [211] used this method to create gyroidal cellulose scaffolds. However, the natural structure of bone tissue is a complex of highly porous multi-layer tissue. Therefore, to fabricate artificial similar bone tissue, the used materials and methods should create highly porous biomimetic materials [123]. Rodríguez et al. [123] used the laser ablation of cellulose acetate electrospun fibres method to create high porosity with extremely small size pores without affecting the surrounding material. The porous mineralised scaffolds significantly increased the attachment and density of bone cells (osteoblast) at the pore sites [123]. Cellulose-based aerogels have been proven to be a promising candidate in tissue scaffold preparation. The mechanical properties of aerogels in the fabrication of designed scaffold for specific tissues allow the target cells to proliferate accordingly. Cellulose/polypyrrole composite aerogels have been fabricated by Shi et al. [212] who evaluated their potential application in nerve regeneration. The aerogel enhanced the adhesion and proliferation of nerve cells (PC12 cells), which were attached and extended longer. The high porosity of the cellulose-based aerogel provides high oxygen permeability and accelerates the exchange of metabolic requirements, which has been necessary for the growth and proliferation of cells leading to enhanced cell activity, better adhesion and increased proliferation and number of cells [213]. Chemical modification to enhance the interaction of cells with scaffolds and integrin-based attachment has also been proposed [214,215]. Nicole Pircher et al. [216] fabricated biocompatible cellulose-based aerogels of dual-porosity. The aerogel scaffolds exhibited high porosity and excellent mechanical properties that enhanced the spread and proliferation of growing fibroblast cells. Li et al. [106] prepared various aerogels from pure cellulose nanocrystals with the direct ink write technique. The technique was used to control the 3D structures of the scaffolds and the inner pore architecture. 3D printing quality can even be improved by increasing the concentration of cellulose nanocrystals and the printing resolution [106]. Various researchers have focused in the past few years to the functional analysis of essential biomarkers and the use of different scaffolds includes cellulose based scaffolds to direct the development of engineered 3D tissue models and organoids. The labelling of nanocellulose with fluorescent probes is of great interest as bio-markers and in sensor applications. Navarro et al. [217] converted cellulose nanofibrils into fluorescently labelled probes, which proved to be viable biomarkers and allowed for fluorescence-based optical detection of CNF uptake and distribution in organisms such as crustaceans. Donnell et al. [218] used the cellulose-binding domain (CBD) for designing biosensor scaffolds. The authors aimed to use these scaffolds to measure pH and Ca^+2^ gradients using fluorescence intensity and lifetime imaging detection modes. The use of cellulose-based scaffolds in diagnostic and biosensing has recently gained more attention. Kim et al. [219] evaluated the potential use of a cellulose-based patch for non-invasive monitoring of interstitial fluid (ISF) glucose levels. The authors used electrospun cellulose/β-cyclodextrin (β-CD), which immobilised the GOx enzyme that can detect the epidermal glucose level. Table 9 shows a summary of the micro- and nanocellulose-based scaffolds in medical applications. Due to the recent advances in tissue engineering, regeneration medicine, and material science, great progress has been done in the development of many fabrication techniques and real cellulose-based scaffolds. Further attention should be given to other applications such as injectable scaffold delivery systems, biosensors, and diagnostic chips. Currently, tissue engineering only plays a small role in biomedicine, providing skin grafts when required, supplementary bladders, or small arteries. The increase in the intensity of accidents and population call for more researches to develop the current and new fabrication techniques. Many organs have been successfully grown in labs, but the use of tissue engineering remains in its infancy and is extremely costly. It has been proposed that the advancement in material development will result in a reduction in the cost of artificial organ development. Cellulose based tissue-engineering scaffolds have been proposed to solve the challenge of availability and affordability of several medical issues. The optimal cell source, scaffold design, in vitro bioreactors, the use, and development of microfabrication technology to create vascularised tissues and organs are still being investigated.

## 5. Conclusions

This review critically analysed the fabrication techniques of micro- and nanocellulose-based scaffolds. Various techniques have been critically reviewed for the fabrication of novel micro- and nanocellulose-based scaffolds with a balance between the mechanical properties and porous architecture that is sufficient to allow cell infiltration and proliferation. The future of scaffold fabrication techniques seems to be in the direction of the rapid prototyping of complex tissue and organs. Based on this review, the cellulose-based scaffold materials developed thus far have 3D frameworks with a high water uptake, tuneable functionalities, physicochemical, and mechanical properties. In addition, it is sustainable and available at low costs. This outstanding set of favourable characteristics offers almost endless possibilities of its application in tissue engineering. The report in this study showed that micro- and nanocellulose-based binary and ternary blends could effectively function as tissue engineering scaffold materials in direct cellular repair and damaged tissue regeneration. However, more studies in grey areas such as toxicity, in vivo, and non-enzymatic degradation of cellulose are needed. Furthermore, scaffold network development of a cellulose-based scaffold using high tech rapid prototyping is needed for the fabrication accuracy of a complex-shaped scaffold. The increase in the number of accidents and population call for more research to improve and develop new fabrication techniques. This will meet the demand for the effective treatment of severely damaged tissues and the high expectation on the development of tissue engineering technology.

## Figures and Tables

**Table 1:** Differences between plants and bacterial cellulose.

Properties	Plants Cellulose	Bacterial Cellulose	References
Purity	Moderate to low	High	[31]
Crystallinity degree	54–88%	65–79%	[32]
Degree of polymerization	Ranged from 500–15,000	800–10,000	[33]
Availability	Highly available	Limited	[34]
Industrial-scale production	Limited	Very limited	[34]

**Table 2:** Material and process of microcellulose materials.

Material	Preparation Process	Particle Size	References
MFC	Acid hydrolysis	9–16 μm	[5]
MCC	Treatment of urea/NaOH	0.05–0.6 μm	[52]
MCC	Enzymatic hydrolysis	0.01–200 μm	[53]
MCC	Catalytic hydrolysis	8.68–31.1 μm	[54]
MFC	Alkali degumming process	0.25–0.30 µm	[47]
MCC	Acid hydrolysis	20 to 374 µm	[43]

**Table 3:** Chronological summary of the research trends on microcellulose-based materials.

Researchers	Progress	References
Payen (1839)	The first-time isolation of cellulose as the principal constituent of wood.	[56]
Schonbein (1845)	The first invention of cellulose esters.	[57]
Ranby (1949)	The first production of microcellulose and nanocrystals with acid hydrolysis of cellulose fibres dispersed in water.	[58]
Ranby (1951)	First synthesised colloidal suspensions of cellulose with acid-catalysed degradation of cellulose fibres.	[23]
Mukherjee et al. (1952)	First TEM images of cellulose materials.	[59]
Battista and Smith (1955)	Microcrystalline cellulose first discovery.	[60]
Colvin et al. (1960)	Formation of micro-fibrillated cellulose in suspensions of *Acetobacter xylinum.*	[61]
Halliwell et al. (1965)	Soil micro-organisms cellulolytic enzymes to re-precipitate cellulose and preparing it by hydrolysis of fibrous cotton.	[62]
Heyn et al. (1966)	Extensively study of the microcrystalline structure of cellulose in cell walls of plants fibres as revealed by negative staining of sections.	[63]
Toshkov et al. (1976)	Development of various method to produce microcrystalline cellulose.	[64]
Kobayashi and Shoda (1992)	First full chemically synthesised cellulose (non-biosynthetic path).	[65]
Revol et al. (1998)	Development of cellulose-based solidified liquid crystals for various optical applications.	[66]
Nakagaito & Yano (2004)	Applying of cellulose microfibril for semi-structural applications	[67]
Kulpinski (2005), Viswanathan et al. (2006)	Electrospinning of pure cellulose.	[59]
Henriksson et al. (2007)	Preparation of micro-fibrillated cellulose nanofibres with an environmentally friendly method for enzyme-assisted.	[68]
Nyström et al. (2010)	Development of nanocellulose polypyrrole composite based on micro-fibrillated cellulose from wood.	[69]
Shao et al. (2015)	Use of micro-fibrillated cellulose/lignosulfonate blends hydrogel rheology on 3D printing.	[70]
Alavi et al. (2019)	Modifications of microcrystalline cellulose for antimicrobial and wound healing applications.	[71]

**Table 4:** Nanocellulose material preparation technique.

Material	Preparation Process	Particle Diameter	Reference
Cellulose nanofibre	Ball milling	100 nm	[86]
Cellulose nanofibre	Electrospinning	70 to 130 nm	[82]
Nanocrystalline cellulose	High-intensity ultrasonication	10 and 20 nm	[87]
Cellulose nanofibre	Blending Method	15–20 nm	[81]
Nanocrystalline cellulose	Acid hydrolysis	10 nm	[88]
Cellulose nanofibre	Twin-Screw Extrusion	30 nm	[89]
Nanocrystalline cellulose	Acid hydrolysis	85 nm	[90]

**Table 5:** Chronological summary of the evolutionary research trend on nanocellulose-based scaffold materials.

Researchers	Progress	References
Ranby (1949)	The first production of microcellulose and nanocrystals with acid hydrolysis of cellulose fibres dispersed in water.	[58]
Turbak and Herrick (1983)	First isolation of nanofibrillated cellulose with mechanical homogenisation of wood.	[95]
Favier et al. (1995)	The first report demonstrating the reinforcing effect of cellulose nanocrystals.	[59]
Azizi Samir et al. (2004)	Isolation of cellulose whiskers reinforced nanocomposites from an organic medium suspension.	[96]
Svagan et al. (2007)	Preparation of cellulose nanofibres bio-foams from wood pulp-based on amylopectin-rich potato starch.	[97]
Henriksson et al. (2008)	Development of Nano-paper from cellulose nanofibre suspensions.	[98]
Fang et al. (2009)	Fabrication of hydroxyapatite/bacterial cellulose nanocomposite scaffolds for the cultivation of human bone marrow stromal cells.	[99]
Rosa et al. (2010)	Isolation and characterisation of cellulose nanofibre from coconut husk fibres.	[100]
Crotogino (2012)	First pilot plant for cellulose nanomaterials production by Innventia in Sweden.	[101]
Dugan et al. (2013)	Development of bacterial cellulose scaffolds and cellulose nanofibre for tissue engineering applications.	[102]
Zhou et al. (2013)	Development of electrospun cellulose nanocrystals-based scaffolds for bone tissue engineering, reinforcing maleic anhydride grafted PLA.	[103]
Yang et al. (2015)	Fabrication of cellulose nanocrystal-based aerogels as universal 3D lightweight substrates for supercapacitor materials.	[104]
Liu et al. (2016)	Development of nanocellulose scaffolds with tunable structures to support 3D cell culture	[105]
Li et al. (2017)	3D printing of many aerogel structures from pure cellulose nanocrystal with direct ink writing technique.	[106]
Apelgren et al. (2019)	In vivo formation of human cartilage in 3D bio-printed constructs with a novel bacterial nanocellulose bio-ink.	[107]

**Table 6:** Conventional and rapid prototyping techniques for scaffold fabrication of micro- and nanocellulose-based materials.

**Part A: Conventional Techniques for Scaffold Fabrication**			
**Technique**	**Operational Condition**	**Principal**	**Reference**
Freeze-drying	Pre-freezing −20 °C for 12 h, then freeze-drying at −80 °C for 48 h	Using aqueous crystals instead of an organic solvent and does not require high temperatures.	[120]
Solvent casting and practical leaching	70 °C to dissolve the salts	Different solvents and salt particles used, which then evaporated leaching out the salts.	[121]
Gas foaming	CO_2_ at 65 bar, 70 °C, the processing time of 1 h	High temperature and organic solvents used in dissolving & inert gases to pressurise modelled it until it saturated or full of gas bubbles.	[122]
Electrospinning	High voltage power supply	Charged threads of polymeric solution or polymer melt are drawn by high voltage electricity.	[123]
Thermal-induced phase separation	Dissolved for 8 h at 70 °C with 300 rpm magnetic stirring, then water evaporation at 100 °C.	Alteration of temperature to force phase separation of the polymer solution, and then dried used to form a nanoscale fibrous network.	[124]
**Part B: Rapid Prototyping Techniques for Scaffold Fabrication**			
**Technique**	**Operational Condition**	**Principal**	**Reference**
Stereolithography	UV-light (365 nm, 1500 W) for 40 s, then −80 °C for 12 h followed by freeze-drying at −68 °C for 48 h	Layer-by-layer printing of photosensitive liquid of polymer with an ultraviolet laser until a 3D scaffold fabricated.	[125]
Selective laser sintering	Laser scan speed 39.8 mm/s, laser power ranged from 1.5 to 3.5 W.	The selective laser used to sinter powdered material in thin layers until a 3D scaffold fabricated.	[126]
Solvent-based extrusion free-forming	Heating temperature of 170 °C and air pressure of 80 psi	Discharging of liquid to solid transition through solvent evaporation in the presence of a binder.	[127]
Bioprinting	3D printing was at 70 °C, then freeze to −70 °C for 12 h followed by freeze-drying.	Fabrication of layer-by-layer, of selective powdered material, then taking out the unbound powder, yielding a complex 3D scaffold.	[128]
Fused deposition modelling	Printing temperature was 210 °C.	Layer-by-layer deposition of polymeric materials extruded through a nozzle to create 3D multiple layers scaffolds.	[129]
Injection moulding	At room temperature	Special moulding machine used to melt and inject the polymeric material into the mould, where it cools and solidifies into the final part.	[130]

**Table 7:** Comparison between conventional and rapid prototyping techniques for scaffold fabrication.

Functionality	Conventional Techniques	Rapid Prototyping Techniques	Reference
Scaffold development time & computer aid	Relatively slow without computer aid	Rapid preparation with computer-aided development	[152]
Manpower or technicians requirements	Require technicians and more manpower needed	Minimize manpower due to computer-controlled fabrication	[147]
Scaffold homogeneity	Not able to develop homogeneous structures	Homogeneous structures can be easily developed	[153]
Control the internal microstructure of scaffolds	Difficult or able to control the internal structures	The internal structures can be easily controlled	[154]
Scaffold porosity	Irregular pores shape and insufficient interconnectivity	Regular and interconnected pores	[155]
Effects on scaffold cytotoxicity	More effect on cells due to solvent residue	Less effect on cells, no solvent residue	[153]
Ability to design accurate & desirable shapes	Difficult to prepare the desirable shape	Easy to prepare even complex shapes	[156]
Cost of production	High cost of production	Low cost of production	[141]

**Table 8:** Research summary of toxicology analysis of micro- and nanocellulose-based materials.

Material	Experiment	Conclusion	Reference
Microcrystalline Cellulose (MCC)	Cytotoxicity and viability evaluation.	No sign for cytotoxicity was observed.	[172]
micro fibrillated cellulose (NFC)	Viability and cytokine of mouse and human cells	Not cytotoxic and does not cause any effects to the cells.	[173]
MFC/ collagen–hydroxyapatite (Col/HA) composite	Viability and proliferation of cells with MTT assay	The composite has been not cytotoxic, biocompatible and safe to cells.	[174]
Microcrystalline cellulose	Haemolytic assay	No cytotoxicity was observed.	[175]
Microcrystalline cellulose/chitosan composite	Cytotoxicity, Thrombogenesis, and haemolytic evaluation.	The composite showed neither cytotoxicity nor thrombogenicity.	[176]
Cellulose-based particles	Viability of the probiotic bacteria assessment.	Generally presented a low toxicity profile to the cell line.	[177]
Cellulose nanofibres	Eco-toxicological and feeding experiments to *Daphnia Magna*.	Low toxic potential to filter-feeding organisms and low expected environmental risks.	[178]
Cellulose nanofibres	Cytotoxicity, skin, and eye irritation tests.	Significantly induced cytotoxicity But not induce skin and eye irritation on 3D models.	[179]
Cellulose nanofibrils	Cytotoxicity and viability of fibroblast cells	No, exert acute toxic phenomena on the cells was observed.	[180]
Cellulose nanocrystals	Cytotoxicity and viability evaluation.	No sign for cytotoxicity was observed.	[181]
Cellulose nanocrystal/silver nanorod	In vitro cytotoxicity of against multiple eukaryotic cells.	No effect on eukaryotic cells was observed.	[182]
Cellulose nanocrystals	Cytotoxicity and viability with the MTT assay.	Cell viability slightly decreased with increasing in CNC concentration	[183]

**Table 9:** Summary of selected applications of micro- and nanocellulose-based scaffolds.

Material	Application & Advantages	Reference
Collagen/oxidized MCC	Microcellulose improved the haemostasis of the scaffolds without affecting its cytotoxicity.	[220]
MFC/gelatine nanocomposite films	Improved strength and flexibility of the films, which could be used in drug delivery.	[221]
Micro-Nano structures of cellulose-collagen	Significantly enhanced the uniform and distribution of cells, with good mechanical properties, may serve as an alternative material platform for bone regeneration.	[222]
MFC/carboxymethyl chitosan hydrogel	The strength, porosity and the work of fracture increased, providing a promising platform for tissue engineering scaffold.	[223]
Alginate/CNF scaffold	Use in tissue engineering. CNF enhances mechanical properties and makes it possible to tailored porosity and swelling behaviour.	[224]
Collagen/CNF hydrogel scaffold	The addition of CNF to collagen scaffold improved its mechanical properties with no effect on cell viability.	[225]
PLA/CNF composite membrane	CNF improved the crystalline ability of the membrane, thermal stability and mechanical properties. Hydrophilicity was also increased.	[226]
NCC/gelatine/hyaluronic acid composite hydrogel	NCC enhanced rheology and swelling results and the other properties. The cells attached, grew, and proliferated better than the control, giving the composite a great potential for the skin wound repair.	[157]
Double crosslinking CNF hydrogel scaffolds	Wound healing and tissue repair. Increase in the rigidity of scaffold enhances cell proliferation.	[149]
